# Recalled attributes of parents with Alzheimer's disease: relevance for caregiving

**DOI:** 10.1080/21642850.2014.971800

**Published:** 2014-10-29

**Authors:** David A. Chiriboga, Yuri Jang, Victor Molinari, Giyeon Kim, Jung Eun Ko

**Affiliations:** ^a^ Department of Child and Family Studies, Florida Mental Health Institute, University of South Florida, Tampa, FL33612, USA; ^b^ School of Social Work, The University of Texas at Austin, 1925 San Jacinto Blvd., D3500, Austin, TX78712–0358, USA; ^c^ School of Aging Studies, College of Arts and Sciences, University of South Florida, Tampa, FL33612, USA; ^d^ Center for Mental Health and Aging and Department of Psychology, The University of Alabama, BOX 870315, Tuscaloosa, AL35487, USA; ^e^ Department of Elderly Welfare, Kyung Hee Cyber University, Seoul130-739, Republic of Korea

**Keywords:** Alzheimer's disease, quality of life, stress, affective responses

## Abstract

Health psychology has long been involved in studies of factors that lead to more effective caregiving. Drawing on the theory of distributive justice, the underlying hypothesis of this paper was that perceptions of what a demented parent was like, prior to becoming ill, influence an adult child caregiver's provision of care, as well as the caregiver's own well-being. A secondary question dealt with the nature of retrospective ratings by caregiver informants. The sample consisted of triads of two adult children (*N* = 385) and a parent (*N* = 201) diagnosed with Alzheimer's disease, although in a few instances only one adult child was interviewed. Both retrospective and current ratings of the parent were made by caregivers, who were administered a semantic differential instrument twice over a 10-month period. Comparison of ratings from first and second interview waves suggested that perceptions of what a parent was like, prior to the onset of dementia, were more stable over time than perceptions of what the parent was currently like, at each interview. Ratings of premorbid attributes were more strongly related to ratings of the present for those parents who displayed the least evidence of cognitive decline. Regression analyses supported the hypothesized relationship between adult children's perceptions and both provision of care and well-being variables. Results have implications for projections of caregiver burden and for placement into long-term care.

## 1. Background

Representing approximately 44% of the 15.5 million caregivers to persons with Alzheimer's disease (AD), adult children comprise not only the largest single group of those who provide care to older family members but also the group reporting the greatest caregiver burden (Alzheimer's Association, 2014; Conde-Sala, Turro-Garriga, Vilalta-Franch, & López-Pousa, 2010; Mack & Thompson, 2005; Reed et al., 2014). With the dramatic increases in life expectancy over the past 60–70 years, especially among those in poor health, the assumption of responsibilities for the well-being of an increasingly dependent parent has in fact become a normative expectation of the middle years (Fiske & Chiriboga, 1990; Turner, Killian, & Cain, 2004). These caregiving responsibilities often come at considerable personal cost, especially when the dependency-creating situation is a cognition-impairing disease such as AD (Piercy et al., 2013; Shin, Carter, Masterman, Fairbanks, & Cummings, 2005).

Just why caregiving to the cognitively impaired is associated with increased burden has been the subject of many studies. The bulk of research has focused on the situation currently being faced by caregivers. Results generally indicate that when dealing with a cognitively impaired family member, the often non-stop time demands, management of complex medication regimens, violent and disruptive behavior, and competing demands from work and family underlies much of the burden (Acton, 2002; Garland, Dew, Eazor, DeKosky, & Reynolds, 2005; Hall et al., 2014; Reed et al., 2014; Schultz, O'Brien, Bookwala, & Fleissner, 1995). In the present paper we take a different focus. Rather than focus on the objective demands imposed by the care recipient's health, we consider the potential impact on caregiver behavior of a care recipient's pre-diagnosis personal attributes, as perceived by the caregiver.

This focus derives from a central premise of the theory of distributive justice: that people make decisions about who receives benefits and help based on whether the recipient of help is seen as deserving that help (Lerner, 2002; Lerner & Clayton, 2011). In the instance of caregiving to a parent, it would follow that a parent who in the past, prior to onset of the AD, was seen more positively would be viewed as a person deserving of care. An implicit corollary to that premise is that providing care to someone seen as deserving of care should reduce the emotional burden of care.

It should be recognized that distributive justice is a variant of exchange theory. While the latter emphasizes that individuals are guided by the desire to minimize the social or fiscal cost of a situation while at the same time maximizing profits (Tyler, Boeckman, Smith, & Huo, 1997), distributive justice and the associated justice motive propose that, when seeking to understand an individual's transactions with others, rules of entitlement play a more central role than profit and cost considerations. The general notion is that when people are perceived as deserving of care they are then more likely to be perceived as entitled to that care. Lerner and Clayton (2011) and others (Baumert & Schmitt, 2012; Walster, Walster, & Berscheid, 1978) have suggested that even during early childhood individuals develop conceptualizations of how things should happen in their world. Instead of profit maximization or cost minimization, the guiding question becomes “Who is entitled to what from whom?”

In the case of an individual with AD, it is hypothesized here that the level of deservingness may depend on current perceptions of what the individual was like prior to onset of the disease (which may or may not reflect what the parent was actually like). To examine the relevance of distributive justice theory for the behavior of adult child caregivers the present investigation drew on techniques developed in an emerging line of research. The latter deals with the implications of pre-diagnosis personal attributes among individuals with some form of dementia. Most of the research has focused on the implications for the individual's subsequent emotional and behavioral problems (e.g. Dawson, Welh-Bohmer, & Siegler, 2000; Meins, Frey, & Thiesemann, 2000).This research is of course made difficult by the near impossibility of interviewing the caregiver-to-be at a time before the parent began exhibiting the signs and symptoms of dementia. For this reason, research has been forced to rely almost completely on retrospective evaluations by caregiver informants. Such reliance has methodological pitfalls. In particular, retrospective accounts may suffer from what is called retrospective bias: they may be heavily influenced by the present relationship and condition of the loved one with AD, the current burden of care, or by the caregiver's perceptions of the disease.

In addition to the problem of retrospective bias, few studies have ascertained the test–retest reliability of retrospective evaluations of the premorbid condition, a potentially fatal flaw when the ratings are being used as a means of predicting downstream behavior of the patient. Of those few that do investigate reliability, however, results from studies with both relatively small sample sizes (under 50), and larger samples of 200 or over consistently report that premorbid personality attributes are both reliable and predict later behavior of the patient (Archer et al., 2006; Meins et al., 2000; Siegler, Dawson, & Welsh, 1994).

The present study is based on caregiver evaluations of a parent with probable AD, and examines the implications of these evaluations for caregiving activity, the burden of caregiving, and general well-being of the caregiver. Drawing from the existing literature two hypotheses guided analyses. The first hypothesis was that caregiver perceptions of what the parent was like in the past would be stable over time. The second hypothesis was that a caregiver's perception of the parent's past attributes would be associated with both the caregiver's provision of care and the caregiver's own well-being. This second hypothesis drew upon theories of distributive justice. These theories and associated research suggested that caregiving would generally be perceived as less burdensome if the recipient is viewed as one deserving of care (Lashewicz, Manning, Hall, & Keating, 2007; Whitbeck, Hoyt, & Huck, 1994). Because the adult children were providing to parents who were obviously not currently responsible for their behavior this study focused on evaluations of what the parent was like in the past.

## 2. Methods and procedures

This investigation employed data from a larger study whose overall focus was on how adult children dealt with the task of providing care to a parent with AD. That study employed a two-wave panel design in which face to face interviews were conducted twice over approximately 10 months. The interval between waves was selected in order to minimize sample attrition while still allowing enough time for progression of cognitive deterioration. Family units were targeted for interviews, with each unit generally consisting of two adult children who both provided care to a parent with a diagnosis of AD, as well as the parent. Data from the parent interviews were not included in the present investigation.

### 2.1. The sample

The sampling frame was targeted on AD patients and their adult children, and was intended to capture potential subjects from all sources that were identified by the research team as existing in a six county area in Northern California. Two strategies were used for recruitment. The first strategy was to identify parents with a diagnosis of AD, and who were reported to have at least two adult child caregivers. The parents consisted of persons aged 60 and over who had received a diagnosis of AD and who were (a) geriatric clients who had sought services from at least one of two hospitals, two clinics, three community health centers, or six home health-care agencies; (b) residents of board and care facilities; and (c) residents of skilled and intermediate care facilities. The second strategy was to identify adult child caregivers who belonged to either of two AD associations serving the catchment area, and who were reported to have at least one sibling who also was providing care to the parent.

Agency and programs were asked to screen for families that met sampling criteria. Due to concern about confidentiality, we could not contact members of each family unit directly. Instead, letters of invitation with self-addressed return postcards were sent by the programs and agencies to known child caregivers. The latter, if agreeing to participate, furnished the names and addresses of another sibling who also provided care. Of the total of 551 adult children to whom letters were sent, 385 agreed to participate and the remainder (30%) either refused to participate or were unlocatable. In 39 instances, erroneous medical or agency records led to families with only one adult child; it was decided to include these families, for comparative purposes. Wave 2 interviews were conducted with 204 adult children and 134 parents, with attrition due primarily to death of the parent or the family moving out of the area.

The first wave of interviews began in 1985, with the second commencing in 1986. The structured interviews were conducted at a time and place of convenience to the participant, with most taking place at the participant's home. Overall, 201 family units participated. The parents were administered a series of structured instruments by the same nurse specialist at both contacts. At the first interview, 32% of the parents lived alone, 22% lived with a spouse who was not the primary caregiver, 24% lived with a family member or friend, 14% lived with a paid helper or in a board and care facility, and 8% lived in a long-term care facility. About 76% were women, and all had previously been diagnosed with probable AD, a diagnosis subsequently corroborated by our own medical team (a geriatrician, nurse specialist, and psychologist).

The adult child caregivers were interviewed by trained graduate students, with nearly all interviews being conducted by the same interviewer at both contacts. Over 58% of the adult children reported that they were the primary caregiver. The proportion of female caregivers, at 71%, approximates that reported in previous studies (Zarit, Todd, & Zarit, 1986). Ranging in age from 24 to 74 (mean = 55), caregivers averaged over 14 years of education, reported an average family income of $30,000, and were primarily Protestant (54%) or Catholic (20%). Sixty-seven percent were currently married, and 27% currently had children living at home. Relevant to caregiving, 70% had contact with their parent on either a daily or weekly basis, and 58% lived less than an hour's distance from their parent. Approximately 89% were non-Hispanic White Americans and the remainder were African-Americans, Hispanic Americans, and other groups.

### 2.2. Measures

#### 2.2.1. Premorbid attributes

To assess the parent's premorbid attributes a peer rating approach was used. Adult child caregivers were asked to rate their parent's attributes as they were before the onset of AD, using a semantic differential technique. The instrument included 12 adjective pairs: grateful–ungrateful, independent–dependent, upset–calm, happy–depressed, kind–cruel, fair–unfair, cooperative–uncooperative, irresponsible–responsible, poor–rich, strong–weak, generous–stingy, and warm–cold. Each pair was presented as a continuum, with ratings being made on a five-point scale (1 = low end of continuum, 5 = high end). Included in the validating analyses, but not in subsequent regression analyses, was another set of ratings that dealt with parent's personality at the present time. While the Cronbach alpha for the combined 12 items, at .76, was marginally acceptable for exploratory studies (Nunnally, 1970) the decision was made to use the individual items in subsequent analyses in order to better understand the types of items most critical for future research.

#### 2.1.2. Socio-demographic variables

These variables assessed socio-demographic characteristics of both caregivers and parent: Caregiver characteristics were assessed by five measures and included information on age, gender, whether caregiver had children, marital status (coded: unmarried or married), family income, and ethnicity (coded: minority or non-minority). Parent demographics included age, gender, and marital status (coded as married or not married).

#### 2.1.3. Caregiver behavior and well-being

Since it would be impossible to assess in one paper the full range of variables that might be affected by perceptions of parent's past attributes, eight dependent measures were arbitrarily selected that covered not only how the caregiver was handling the demands of caregiving, but also their general well-being. Four measures focused specifically upon behaviors and issues related specifically to caregiving:

(1) *Assistance with Daily Living Scale (AWDLS).* A modification of the OARS Activities of Daily Life Scale (Duke University, 1978) that registered the extent of care provided patients in 14 ADL and IADL areas. Adult children were asked how often they assisted their parent in each area. For example, how often did they make phone calls for the parent, take them to the doctor, help in the preparation of meals, etc. For each item an 8 point rating scale was used, where 1 = “never” and 8 = “more than once a day.”Included was an AWDLS summary score (alpha for the present study = .93).
(2) *Stress experienced* A 4 point rating on how stressful the caregiving experience had been, where 1 = “very stressful” and 4 = “not stressful.”
(3) *Anticipated future* A 4 point rating on how much better or worse the future is anticipated to be, where 1 = “much worse” and 4 = “much better.”
(4) *Caregiver burden* A summary score of the full 21 item Caregiver Burden Interview (Zarit et al., 1986); higher scores indicate greater burden (study alpha = .90).

The remaining four dependent measures dealt with relatively stable aspects of psychological well-being:

(5)  *Depressive symptoms* A 23 item subscale of the Hopkins Symptoms Checklist-90 (Derogatis & Cleary, 1977) was used; higher scores indicate greater depressive symptomatology but are not necessarily indicative of clinical depression (study alpha = .89).
(6)  *Anxiety symptoms* Another subscale of the Hopkins Symptoms Checklist-90; higher scores indicate more symptoms of anxiety but again are not indicative of any clinical state (study alpha = .88).
(7) *Negative affect* A 5 item subscale of the Bradburn (1969) Morale Scale; higher scores indicate more negative emotions experienced during the preceding week (study alpha = .63).
(8) *Positive affect* Another 5 item subscale of the Morale Scale; higher scores indicate more positive emotions experienced during the preceding week (study alpha = .68).

These dependent measures were not necessarily independent of each other. The indicators of psychological well-being were intercorrelated. For example, Negative Affect correlated significantly with depressive (*r* = .66, *p* = .00) and anxiety (*r* = .57, *p* = .00) symptoms, and the symptoms of anxiety and depression were themselves strongly correlated (*r* = .73, *p* = .00). On the other hand, none of the dependent variables specific to caregiving correlated with each other, or with the more psychological indicators, at levels higher than the mid-30s.

### 2.3. Analyses

Initial analyses considered the Wave 1 vs. Wave 2 correlations between caregiver ratings of their parent's attributes both before any dementia was apparent and at the present time. The correlations were followed by a series of hierarchical regression analyses of Wave 1 data, where the four indicators of caregiver behavior and the four indicators of caregiver well-being were regressed on (1) socio-demographic characteristics of the caregiver, (2) socio-demographic characteristics of the parent, and (3) informant ratings on the pre-morbid personal attributes of the parent.

## 3. Results

### 3.1. Stability of personality ratings

The key variables for this investigation consisted of caregiver ratings of what their parent was like before the appearance of signs and symptoms of AD. While our primary interest was in how these caregiver recollections of their parent might influence the caregiver's general well-being as well as caregiving itself, we also wished to examine evidence for or against the argument that retrospective evaluations of the parent persist over time. It was hypothesized that ratings of the past would be more highly correlated over time than would ratings of what the parent was like at the present time, since even during the limited interval between interviews some changes could be anticipated in current personal attributes of the parent. For these reasons, caregivers were asked to rate their parent's attributes, past and present, at both interview contacts.

As shown in Table 1, ratings of the parent's past attributes were significantly and strongly correlated across the two waves of the panel study. The two waves were separated by approximately 10 months and yet the correlations range in magnitude from .51 to .74, with an average of .62. The magnitude of these correlations approximates that found in studies of self-reported personal attributes (Costa & McCrae, 1994; Fiske & Chiriboga, 1990; McAdams & Olson, 2010).

**Table 1.** Correlations between Time 1 and Time 2 for semantic differential evaluations of a parent prior to onset of symptoms (*N* = 199) and evaluations for the present (*N* = 141 to 180)^a^, and between prior and present status for parents in the bottom two-thirds (*N* = 153–199)^a^ or upper one-third (*N* = 104–106) in cognitive status based on Mini-Mental Exam^a^.

	T1 vs. T2 correlations		Prior vs. present correlations	
	Prior to onset	Present status	Lower 2/3 in cognition	Top 1/3 in cognition
Grateful	.60*	.49*	.29*	.39*
Upset	.62*	.52*	.23*	.27*
Independent	.74*	.31*	.10	.18
Happy	.70*	.59*	.20*	.38*
Kind	.58*	.56*	.35*	.57*
Fair	.62*	.58*	.28*	.33*
Cooperative	.55*	.45*	.27*	.40*
Irresponsible	.51*	.44*	.09	.04
Poor	.52*	.57*	.51*	.76*
Strong	.71*	.57*	.30*	.36*
Generous	.62*	.47*	.34*	.55*
Warm	.66*	.47*	.39*	.50*
Average correlation	.62	.50	.28	.39

^a^Sample Ns for these correlations varied; more subjects felt unable to rate parent's current personality, at both T1 and T2.

*Pearson correlation *p* = .00.

Caregivers' ratings of their parent's current attributes demonstrated less stability across the two interview waves. Correlations ranged from .31 to .59, with an average intra-item correlation of .50. A t-test for the difference in magnitude between correlation for parents' items for the past and present indicated that the former were significantly higher (*t* = 3.72, df = 22, *p* = .00). Together these findings suggest that ratings of a parent's attributes in the past are tapping more durable perceptions of the parent; the findings also suggest that the progressive nature of AD modifies how parents are currently viewed, even over a relatively limited period of time.

Additional information came from a comparison of ratings for the past vs. the present. It was hypothesized that there would be greater similarity between ratings of the past and present for parents with less evidence of cognitive decline. To test the hypothesis, correlations between past and present ratings were compared for parents who fell into the top one-third of possible total scores on the Mini Mental State Exam (Folstein, Folstein, & McHugh, 1975), vs. parents who fell into the lower two-thirds.

As also shown in Table 1, ratings of premorbid attributes were more strongly related to ratings for the present for those parents who displayed the least evidence of cognitive decline. These group differences in correlation magnitude were significant (*t* = −1.79, df = 22, *p* = .04). Moreover, the differences are consistent with what would be expected if the retrospective ratings by caregivers do in fact represent relatively lasting or durable impressions: retrospective and current ratings of those parents with less cognitive deterioration would be expected to correlate more highly than those for parents with greater deterioration. Coupled with the finding that the retrospective evaluations were more stable over time than were ratings of how the parent was currently, the results combine to emphasize the durability of impressions about what the parent was like prior to the onset of AD.

### 3.2. Regression of caregiver activities and well-being on predictors

In addition to considering the reliability of the retrospective ratings, another objective of the investigation was to consider whether perceptions about the parent's premorbid attributes might influence the previously described outcome measures: (1) four indicators of caregiving activities and distress about the caregiving situation and (2) four indicators of the caregiver's general well-being. Data from only the first interview were used in the analyses, in order to maximize sample size. Hierarchical regression analyses were computed, with the successive models in each analysis including demographic characteristics of the caregiver, demographic characteristics of the parent, and parents' characteristics. Results indicated that the predictive models, while generating significant results for all outcome measures, included many variables that contributed little or nothing to the final equation. For this reason the analyses were re-run using only predictors that had reached at least trend (*p* = .10) levels of significance in the initial regressions. It is this second set of analyses that will be described. We will begin by considering the regressions where the outcome variables reflected the caregivers' thoughts and activities specific to caregiving.

#### 3.2.1. Assistance provided

As shown in Table 2, all three sets of variables together accounted for 13% of the variance in the amount of assistance provided by the caregiver. Caregiver socio-demographic characteristics accounted for the most variance: minority caregivers, daughters, and those with lower income were more likely to provide assistance. The significant parent socio-demographic characteristic was marital status: caregivers were more likely to provide assistance to unmarried parents. Among the premorbid perceived attributes, children were more likely to provide assistance to parents perceived as dependent and more rich than poor.

**Table 2.** Set statistics and beta weights at entry from hierarchical regression analyses predicting how well the caregiver is doing with caregiving at first interview.

	Assistance provided (*n* = 199)			Current stress (*n* = 278)			Evaluated future (266)			Burden (279)		
	*B*	*R*^2^	▵*R*^2^	*B*	*R*^2^	▵*R*^2^	*B*	*R*^2^	▵*R*^2^	*B*	*R*^2^	▵*R*^2^
SET 1: caregiver		.07	.07***		.04	.04***		.01	.01*			
Women	.10*			−.18***								
Income	−.20***											
Non-ethnic	−.15**			−.08			−.10*					
SET 2: parent		.10	.03***					.04	.03***		.03	.03***
Married	−.17***						−.18***			−.17***		
SET 3: attributes: patient		.13	.03**		.09	.05***		.07	.03***		.14	.11***
Dependent	.11*						−.09*			.13***		
Rich	.11*											
Fair				−.13**						−.14***		
Depressed				−.14***						.13**		
Calm							.15***					
Ungrateful										.12**		

Note: Based on analyses limited to predictors originally contributing at probabilities equal to or below .10. Only results with probabilities of .10 or higher are shown*.*

*p < .05.

**p < .01.

***p < .001.

#### 3.2.2. Perceived stress in dealing with the parent

Four variables together accounted for an overall 9% of the variance in reported caregiver stress. Two were characteristics of the caregiver: women and minority caregivers were less likely to report feeling stressed by the caregiving role. Accounting for 5% of the variance, the other two indictors dealt with perceived attributes of the past: adult child caregivers were less likely to report stress if their parent was rated as having been more fair and happier prior to the development of AD.

#### 3.2.3. Evaluation of the future

The four variables included in the predictive equation accounted for a total of 7% of variance. Minority caregivers tended to be more optimistic about the future, but this variable accounted for only 1% of the variance. Both parent-related sets of variables made significant, albeit relatively small, contributions. Curiously, if the parent was currently unmarried, the adult child caregiver was more likely to be optimistic about the future. Also, caregivers reported a brighter outlook for the future if the parent was perceived as having been more independent and/or a calmer person.

#### 3.2.4. Caregiver burden

No demographic characteristics of caregivers were related to the caregivers' reports of burden, but again the two parent-related hierarchical sets made significant contributions and accounted for 14% of the variance. With regard to parent demographics, having an unmarried parent was associated with greater caregiver burden. The attributes of parents made a particularly strong contribution: four of the attributes were significantly related and together they accounted for 11% of the variance. Viewing a parent as having been more dependent, unfair, depressed, and/or ungrateful was associated with the caregiver reporting greater burden.

### 3.3. Regression of caregiver indicators of well-being on predictors

The dependent variables shown in Table 2 dealt specifically with the context of caring. In a second set of analyses, attention turned to functional qualities that are not linked to any specific context, and which generally exhibit at least moderate stability over time: emotions and psychological symptoms (Costa & McCrae, 1994; Fiske & Chiriboga, 1990).

#### 3.3.1. Depressive symptoms of caregivers

The regression equation accounted for a relatively minor 7% of the variance in caregiver depression. As shown in Table 3, female caregivers and those with lower incomes reported greater depression. Socio-demographic characteristics of the parent did not contribute, but caregivers who viewed their parents as having been “weak” were also more likely to be depressed.

**Table 3.** Set statistics and beta weights from hierarchical regression analyses predicting the general psychological well-being of the caregiver at first interview. Based on analyses limited to predictors originally contributing at probabilities equal to or below *p* = .10.

	Depression (*n* = 199)			Anxiety (199)			Negative affect (*n* = 199)			Positive affect (*n* = 199)		
	*B*	*R*^2^	▵*R*^2^	*B*	*R*^2^	▵*R*^2^	*B*	*R*^2^	▵*R*^2^	*B*	*R*^2^	▵*R*^2^
SET 1: caregiver		.04	.04***								.02	.02**
Women	.16***											
Income	−.13**									.15**		
SET 2: parent											.04	.02**
Married										−.13**		
SET 3:attributes: patient		.07	.03***		.05	.05***		.04	.04**		.06	.02*
Depressed							.22***			−.13		
Cooperative							.11*					
Weak	.16***			.22***								
Dependent												
Kind										.11*		

*p < .05.

**p < .01.

***p < .001.

#### 3.3.2. Caregiver anxiety

Surprisingly, the only hierarchical set to contribute to the prediction of anxiety symptoms was the one tapping the perceived attributes of the caregiver's parent. As was the case in the prediction of depressive symptoms, having a parent perceived as having been “weak” was associated with greater caregiver anxiety. This single variable accounted for 5% of the variance.

#### 3.3.3. Negative well-being of caregiver

While perceived attributes constituted the only variable set significantly associated with Negative Affect, as was the case for anxiety the set accounted for a relatively minor proportion of the variance: 4%. Having a parent viewed as more depressed in the past was significantly associated with greater negative affect on the part of the caregiver, and there was a trend for negative affect to be associated with having been a more cooperative parent.

#### 3.3.4. Positive well-being of caregiver

In predicting positive affect of the caregiver, four variables accounted for an again relatively low 6% of the variance. All three sets contributed, although parent attributes were associated at only the trend level. Having a lower income, having a presently unmarried parent, and viewing one's parent as having been more depressed were significantly associated with lower positive affect; there was also an association at the trend level for adult child caregivers to report lower positive affect if they viewed their parent as more on the kind side of the kind–cruel continuum.

## 4. Discussion and conclusion

As noted in the background section of this article, the primary question to be addressed was whether or not the personal attributes of parents before they develop AD play a role in the burden of caregiving and in the psychological well-being of adult child caregivers. This question draws from the theory of distributive justice, which suggests that whether or not someone is seen as deserving of care should affect the delivery of care, as well as the perceived burden. Results demonstrate that the pre-onset attributes of parents may influence both the perceived burden and distress of caregiving, and the caregiver's psychological health. Additional analyses represented an initial attempt at determining whether the current situation faced by caregivers influenced caregiver ratings of their parents, or whether these ratings showed reasonable stability over time. Here results indicated that ratings of pre-onset attributes of parents showed more stability over a 10-month period than did ratings of the parent's current attributes at the time of each interview. Combined, these results suggest that health providers interested in estimating future caregiving problems and potentials may wish to ask adult child caregivers about family relationships prior to the onset of dementia.

### 4.1. Stability of retrospective reports

Turning first to the question of stability over time, one advantage of the research was not only the relatively large sample of caregivers who provided retrospective reports but also the fact that caregivers were interviewed twice. The repeated interviews provided an opportunity to assess whether ratings of past parent attributes were more stable than ratings of current attributes. The fact that pre-onset attributes were more stable suggests that they may have a more lasting influence on caregiver behaviors. Findings also provided some support for the validity of ratings of past attributes, since it was also found that these ratings were more strongly correlated with present ratings for those parents who displayed the least evidence of cognitive impairment. In other words, the ratings of the past and present attributes of the parent were more highly correlated if the parent had experienced less cognitive decline. This last finding is consistent with the considerable evidence in the literature that AD is associated with progressive changes in personality. The World Health Organization (2004) International Statistical Classification of Diseases and Related Health Problems for example cites the frequent association of personality change with progression of dementia. In the present case, the correlations also suggested that parents who had progressed further in their dementia had changed in their personal attributes.

### 4.2. Relevance of retrospective reports for current activities and well-being of adult child caregivers

With respect to the salience of the premorbid parent attributes for the present situation faced by the caregiver, probably the first and most obvious point to be made is that the regression equations as a whole explained significant – albeit relatively small – proportions of the variations in both caregiver behavior and general well-being. Socio-demographic characteristics of caregiver and parent contributed the least, while ratings of pre-onset attributes of the parent adding from 2% to 11% of explained variance.

The relatively poor showing of socio-demographic indices confirms previous findings that caring, and the burden of caring, by and large reflect the unique circumstances of individuals and their families (Feeney & Collins, 2001; Lerner & Clayton, 2011; Morse, Shaffer, Williams, Dooley, & Schulz, 2012). The significant but relatively small contributions of recalled attributes of the parent, which certainly are sensitive to at least one aspect of family uniqueness, are both encouraging and a signal that more work needs to be done. The contributions are encouraging because results support the hypothesis, based on the theory of distributive justice, that recollections of the parent's past attributes would predict the current well-being of adult caregivers and the latter's caregiving behaviors. For the most part, caregivers who recalled their parent as having more negative attributes were more likely to report greater distress related to the caregiving experience. This is in line not only with theories of justice, but also with early research indicating that for caregivers the present problem, whether it be a dementia or some other debilitating problem, is less important than whether the person is seen as being worthy of care (e.g. Weiner, 1993). On the other hand the relatively small the contributions made by the recalled attributes highlights the need for better measures of what the parent was like.

It is important to note that the strongest contribution of these attributes was in the prediction of caregiver burden, which itself is a salient predictor of a host of negative outcomes for the recipient of care, including elder abuse, mortality rates, and premature institutionalization (Schultz et al., 1995). These findings support the hypothesis that a caregiver's perception of what the parent had been like may help to shape how the caregiver responds to the caregiver situation. The results thus help in putting together the puzzle of caregiving behavior.

Our findings also correspond with the emerging literature on premorbid attachment styles and caregiver experiences. This literature suggests that the pathways to negative caregiver outcomes may involve an intricate relationship between the ongoing behavioral and physical problems of the family member with AD, perceived premorbid personal attributes, and premorbid attachment styles of AD parents. All of these have also been shown to be predictive of caregiver burden and psychiatric symptomatology (Feeney, & Collins, 2001; Morse et al., 2012).

The current results are consistent not only with the “who deserves what” assumptions of the theory of distributive justice, but also with the literature on families in general. For example, many years ago Troll (1988, p. 590) pointed out there are at least three core ideas concerning what underlies good parent–child relationships: “Above all, we value independence. Secondly, we value self-realization or self-fulfillment. But we also value filial obligation and familism.” A parent viewed as having been an unfair or ungrateful person may create a seemingly unwarranted intrusion on the caregiver's quest for personal autonomy and self-fulfillment, a “good” parent may underscore for the child caregiver the importance of filial obligations.

### 4.3. Study limitations

While the significant associations found between recalled attributes of parents and several indicators of caregiver functioning suggest that retrospective evaluations of parents may be useful indicators, the study had several limitations.

#### 4.3.1. The lack of a randomized sample

Due to legitimate agency concerns about providing researchers with their rosters of clients, we had to allow each agency to notify potential participants, who returned postcards if willing to participate. This method eliminated the possibility of a randomized sampling procedure.

#### 4.3.2. Geographic and resulting racial/ethnic limitations

Participants were all from a relatively limited region in northern California that included relatively few members of racial/ethnic groups other than non-Hispanic Whites. For this reason the implications of study findings may not apply to other groups.

#### 4.3.3. Reliance on a new, and single, approach to assessing parent attributes

In the absence of any standard approach to assessing parent attributes, the decision was made to ask about very general attributes (strong vs. weak, kind vs. unkind, etc.), using the semantic differential approach. Future studies may wish to focus on more specific behavior of the parent, such as instances of corporal punishment, whether the parent showed favoritism in dealing with children, or was involved in the homework assignments or hobbies of children. More generally, while this and several other studies have demonstrated the relevance of retrospectively obtained information on the family, especially in the area of social relationships, little is known about the reliability and validity of these variables. The investigation presented here is just one small step toward establishing the scientific merit of retrospective ratings of parent attributes. Indeed, many would consider use of such an approach inherently flawed: that like nations we as individuals are prone to rewrite our pasts to fit our present. In studies like the present one, however, this last general criticism of retrospective data may actually not apply. According to the theory of distributive justice what is most critical is not the reality of what the individual with AD was actually like. Rather, at issue is whether the caregiver currently views the individual as someone who in the past was a good and deserving person.

### 4.4. Conclusion

Regardless of the importance of accurate assessment, given the impossibility of revisiting the past and the difficulty in identifying subjects prior to the onset of AD the use of retrospective measures has distinct appeal. It was encouraging that the present study found levels of stability in retrospective ratings that were comparable when personality is self-assessed. The findings are also consistent with a growing literature confirming the consistency of family premorbid perceptions and the reliability of different informant judgments.

Practical implications can be drawn from these findings. From a clinical perspective, deeply held family perceptions of dependent elders may provide clues about historical attachment bonds across the family network that yield valuable information for treatment planning. For example, they may provide information as to why some patients are institutionalized much earlier than others, despite equal levels of disability and potential caregiver availability. From a systems-of-care approach, our results suggest that screenings which target potentially “at risk” community-dwelling AD patients should consider assessing the perceptions of family members about premorbid attributes of the care recipient.

Finally, the present study adds to the corpus of knowledge on the utility of “collateral” ratings when attempting to understand and care for a cognitively impaired family member. The results presented here demonstrate that perceptions about whether or not a parent was kind or fair or dependent, etc. endure over time and can affect caregiving behavior, including the burnout that may arise from stress and burden. Future studies should explore in more depth how retrospective evaluations by family members can facilitate treatment interventions for persons with impaired cognitive functioning, and for their caregivers.
